# 

**Published:** 1885-05

**Authors:** 


PAGE.

ORIGINAL COMMUNICATIONS:

The Pre-Albuminuric Stage of Chronic Bright's Disease. C. W. Purdy, M. D. .... 379
Mollities Ossium. W. J. Webb .... 414
Neuroses Due to Anomalies of the Eye and Ear. H. Gradle, M. D. .... 430

CORRESPONDENCE—DOMESTIC .... 438

EDITORIAL:

The American Medical Association .... 440
Objective Diagnosis .... 442

SOCIETY PROCEEDINGS:

Chicago Gynecological Society—
The Influence of Cimicifuga Racemosa upon Parturition. J. S. Knox, M. D. .... 444

PAGE.

Some Remarks on the Value of Permanganate of Potash in Amenorrhœa. E. J. Doering, M. D. .... 449

Report of a Case of Hysterectomy for Intra-Mural Myoma. E. C. Dudley, M. D. .... 450

BOOK REVIEWS .... 452

NEWS ITEMS .... 458

ABSTRACTS AND EXTRACTS:

Obstetrics and Gynæcology .... 460
Observations of the Determination of the Hearing Power. W. Laidlaw Purves, M. D. 469

PERSONALS .... 473

LOCAL NEWS .... 474

